# Perceptions of primary care services among Afro-Peruvians in Lima, Peru

**DOI:** 10.1017/S1463423625000076

**Published:** 2025-01-31

**Authors:** Elisa Juárez-Chávez, José H. Villalobos Ruiz, Kelika A. Konda, Dayana Urday-Fernández, María Sofía Cuba-Fuentes

**Affiliations:** 1 Universidad Científica del Sur, Lima, Peru; 2 Universidad Nacional Mayor de San Marcos, Lima, Peru; 3 Universidad Peruana Cayetano Heredia, Lima, Peru; 4 Center for research in Primary health Care, Universidad Peruana Cayetano Heredia, Lima, Peru

**Keywords:** Afro-Peruvian, health systems, healthcare access, healthcare disparities, minority populations, primary health care, public health, qualitative research, patient experiences, patient-provider interaction

## Abstract

**Introduction:**

The Peruvian public healthcare system is characterized by various shortcomings that adversely affect healthcare quality as perceived by the general and minority populations, including the Afro-Peruvian community. This population has demonstrated reduced healthcare access due to discrimination and differential treatment, reflecting broader societal inequities.

**Objective:**

This study explores the experiences and perceptions of Afro-Peruvian individuals regarding the treatment they receive from public primary healthcare providers in metropolitan Lima.

**Methods:**

In-depth qualitative interviews were conducted with Afro-Peruvian individuals recruited from Lima. They were selected based on their responses to a survey conducted in a previous study, which indicated a high or low perception of intercultural adaptation in healthcare. The interviews explored their experiences with healthcare services and their perceptions about their interactions with health providers. The qualitative analysis involved topic coding to interpret the data.

**Results:**

We interviewed 19 Afro-Peruvians, including 15 women and 4 men, ages 26 to 70. The findings reveal that Afro-Peruvians generally experience mistreatment in the healthcare system. In their opinion, this is associated with systemic issues such as poor infrastructure, low salaries, and insufficient time allocated for patient care. Furthermore, participants perceive receiving poor quality and inefficient service not only from providers but also from the system presents difficulties in other processes, such as getting the appointment.

**Conclusions:**

This study highlights significant areas for improvement in the public healthcare system, specifically enhancing the quality of patient care, improving communication, and upgrading healthcare infrastructure to serve the Afro-Peruvian community better. These insights could guide the development of targeted policy recommendations and practical interventions to address healthcare disparities and improve access to quality healthcare services for minority populations.

## Introduction

### Healthcare provision and access

Access to high-quality healthcare constitutes a fundamental right and represents a moral imperative for the nations (UN Committee on Economic, Social and Cultural Rights, 2000). Healthcare services do not have a unique way of delivery, but they can be directly consumed by an individual, whether preventive, diagnostic, therapeutic, or rehabilitative, or they can be delivered through actions that are applied either to collectivities (e.g., mass health education) or to the non-human components of the environment (e.g., basic sanitation) (Murray, C. J., & Frenk, J. 2000). When the provision of health care addresses all the issues of accessibility, affordability, efficiency, and quality, it contributes towards the improvement of the health equity of the population that it serves (Syafinaz *et al.* 2016).

On the other hand, access to healthcare is the opportunity to reach and obtain appropriate healthcare attention (Levesque *et al.*., 2013). Access is not determined by a sole element, but is affected by elements that are supply-side (features of the services and systems), demand-side (features of populations), and/or to process factors (Harris & Russell, 2013).

More importantly, access to health care does not only refer to receiving treatment for specific disease but also to a more holistic term that includes the identification of healthcare needs, seeking healthcare services, reaching healthcare resources, and being offered appropriate healthcare services based on needs, and to obtain or use health care services.

Even when access to health care should be universal, as it mandates its human rights category, some groups are particularly affected by the limited access to health care. Such groups include people with disabilities, members of the LGTB community, migrants, Afro-descendants, among others (Wilson & Yoshikawa, 2007; Rodríguez Gatta *et al.*., 2024; Schwarz *et al.*., 2022; Feitosa M. *et al.*., 2021; Khatri & Assefa, 2022). In settings where access is limited, such as low- and middle-income countries, these groups are particularly affected and become even more vulnerable.

Particularly in Latin America, the quality of health services has proved to be a severe problem for the population. According to a report from 2022 to 2023, only one-third of health service users reported receiving high-quality care (Roberti J *et al.*., 2024). This perception of suboptimal quality in healthcare is intricately linked to a range of barriers encountered by individuals when accessing health services. Key impediments include insufficient acceptability (38%), limited accessibility (29%), inadequate contact (22%), and restricted availability (11%) (Hirmas A *et al.*., 2013). Specifically, among these identified barriers, issues manifest as fear or embarrassment related to seeking care (7.6%), distrust in healthcare providers and prescribed interventions (6.1%), and the influence of social stigma, beliefs, and myths (6.1%) (Hirmas A *et al.*., 2013). These multifaceted challenges collectively perpetuate the perception of healthcare services as inadequate (Hirmas A *et al.*., 2013).

### Access to healthcare in Peru

In Peru, difficulties in accessing health services have led to a decrease in their use and increased use of pharmacies (used as consultation places) in the presence of symptoms perceived to be uncomplicated. According to a report from the Peruvian National Institute of Statistics and Informatics, in the last quarter of 2021, about one-third of the population (34.7%) with a health issue sought health care. Among them, almost half (46.4%) sought care at a pharmacy or drugstore, 26.8% went to a Ministry of Health (MINSA, Spanish acronym) facility. This was followed by 15.3% who visited a private clinic, and 9.5% sought care at EsSalud facilities (the health system for individuals with formal employment) (INEI 2023).

Studies conducted in the country demonstrate that the main elements affecting access to healthcare in Peru are associated to the healthcare facility (including its infrastructure), distance to the user, and available human resources (HR) (Houghton *et al.*. 2023). Even when these elements affect all the population, minority groups have been proven to be even more affected due to (Hernadez-Vasquez *et al.*., 2022). For example, Afro-Peruvians, who represent 3.6% of the Peruvian population according to the 2017 census (INEI, 2018), have been demonstrated to have reduced access to healthcare due to substandard service quality and limited accessibility (Defensoria del Pueblo, 2011), leading to a significant percentage of them choosing not to seek healthcare services when they have an illness or emergency.

Being this said, we considered it essential to describe the elements that might be influencing patient–doctor interaction and how it is perceived by afro-Peruvian population that use them. While this study is focused on Afro-Peruvians, there are many marginalized groups, and health service research should explore the treatment and experiences of such groups to work toward health services are adequate for all users. Thus, the study intends to provide a basis for improving health services for marginalized groups.

## Methods

### Context

The Peruvian healthcare system should be designed to guarantee the provision of healthcare services and improve the population’s health and quality of life (MINSA, 2020). However, in reality, it is a fragmented and segmented system (Videnza Consultants, 2020), where access to care depends on one’s socioeconomic and employment status, as there are subsystems, each with its own financing and provision processes, additionally to a high us of out-of-pocket expenditures. The two most important systems funded by state resources are the Ministry of Health (MINSA SIS), which is financed through taxes and primarily serves economically disadvantaged individuals; and EsSalud, the employee health insurance system, which is funded through a payroll tax on the salaries of formally employed individuals. The insufficiency or poor efficiency of these services has led to the creation of public–private alternatives for care provision, such as municipal services (offered at low prices and with some level of municipal funding) and various types of private services aimed at meeting the population’s healthcare needs.

### Study design and setting

This study employed an exploratory and descriptive design, intended to construct ethnographic knowledge by applying in-depth interviews and their corresponding analysis. The development of tools was grounded in key dimensions of the barriers and facilitators for accessing as well as exploration of their experiences in the health services. The study took place in Lima, the capital of Peru, home to the country’s largest Afro-Peruvian population. According to the 2017 census, almost 27% of this population lives in Lima (15), with no other of the 25 regions reaching this percentage.

### Study participants

The current study was complemented by a quantitative survey conducted among Afro-Peruvians from six Peruvian cities, including Lima, to explore their perception regarding the intercultural adaptation of health services for Afro-Peruvians. The survey results have been published previously (Contreras *et al.,* 2024). Among the individuals surveyed in Lima, a location chosen for proximity to the study team and for having the largest Afro-Peruvian population, 19 individuals were selected to be interviewed for the current study. The selection criteria for the qualitative interviews were based on their survey responses, choosing participants from the upper (perception of high intercultural adaptation) and lower extremes (perception of low intercultural adaptation). Since we did not identify any relationship between the questionnaire score on intercultural adaptation of health services and perceptions of the treatments received, we will not provide further reflections on this characteristic in our analysis.

The Institutional Ethics Committee for Research of the Universidad Peruana Cayetano Heredia approved the conduct of this study (Study number 522-44-22).

### Qualitative interviews

In-depth interviews were conducted with individuals self-identifying as Afro-Peruvian, residing in metropolitan Lima, who have utilized state-run healthcare services (specifically MINSA SIS or EsSalud) at the primary care level. The fieldwork was carried out between January and April 2023. Thirteen interviews were conducted face-to-face, while six were conducted by telephone based on participant request. The in-depth interviews took approximately 45–60 minutes and were performed by an anthropologist (JVR) with extensive qualitative research experience. No prior relationship was established between this researcher and the participants. When the interviews were done in person, compensation was provided to cover transportation expenses, and a snack was provided. No compensation was provided for the phone-based interviews. All the interviews were done in Spanish and audio recorded.

The interview guide included open-ended questions focused on understanding participants’ experiences with healthcare services and their perceptions about their interactions with health providers. Even when participants reported using private and public healthcare services, we focused the interviews on their experiences and perceptions of public services.

### Analysis

The analysis team comprised two of this article’s authors (first and second author). Before analysis, the audio files of the interviews were transcribed verbatim. Together, the analysis team constructed and discussed an initial version of the codebook with codes based on elements included in the interview guide, following a deductive coding approach. Once the initial codebook was established, one of the transcriptions was analyzed by the analysis team to assess the codebook’s utility and to ensure coding agreement. After coding the first transcript, the analysis team met and discussed the codes’ meanings prior to additional analysis. Subsequently, the second author coded the remaining transcriptions. Codes were applied to interview transcripts using Excel.

**Figure 1.**
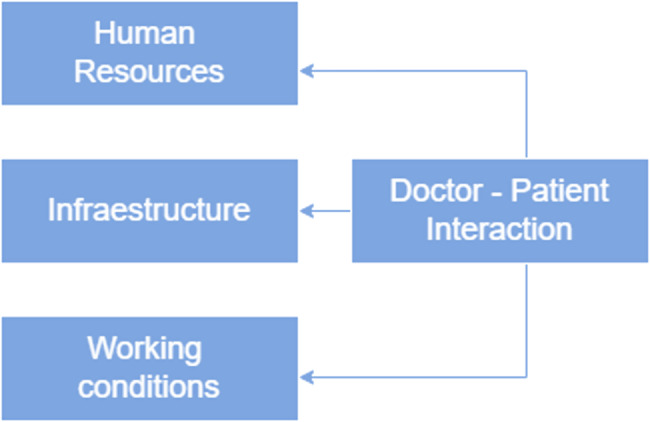
Framework for presenting results.

A constructivist approach was used to include additional codes as emerging topics were elucidated during analysis. Regular meetings were held between the first and second authors to discuss new codes. These codes were mainly focused on experiences in healthcare services and perceptions of discrimination and personnel treatment (both directly associated with human resources), infrastructure conditions, and the working conditions of health providers. Those elements were grouped to build a framework (see Figure 1). After coding, the analysts compared the responses by code to identify similarities and differences across participants to evaluate which were the most frequently mentioned problem.

Additionally, a documentary review was conducted to describe the healthcare system in Peru and provide a better context for understanding our results. The whole analysis process was done in Spanish; only the quotes selected for use were translated into English.

## Results

Nineteen Afro-Peruvian individuals, fifteen women and four men aged 26–70 years were interviewed for this study and reported using various healthcare systems (see Table 1). According to their testimonies, their decision to use one healthcare system over the other was not solely dependent on the individual’s insurance. Despite having EsSalud or insurance from the Ministry of Health (SIS), several interviewees mentioned using private or municipal services, which they pay for out-of-pocket. This choice was mainly based on perceptions of quality, time, and the relative cost/benefits of the public versus private systems.

**Table 1.** Primary characteristics of the interviewees

Gender	Frequently used health system	Age	Chronic disease**
Female	ESSALUD	Between 36 and 40	No
		Between 46 and 50	No
		Between 66 and 70	No
		Between 51 and 55	Yes (Hypertension and diabetes)
		Between 36 and 40	No
	ESSALUD and PRIVATE	Between 61 and 65	Yes (Diabetes)
	MUNICIPAL/ESSALUD	Between 46 and 50	No
	PRIVATE**	Between 41 and 45	No
		Between 66 and 70	Yes (cancer)
		Between 31 and 35	No
		Between 41 and 45	No
	MINSA	Between 31 and 35	No
		Between 36 and 40	No
		Between 26 and 30	No
		Between 36 and 40	No
Male	MUNICIPAL/MINSA	Between 31 and 35	No
	PRIVATE*	Between 61 and 65	No
	MINSA	Between 36 and 40	No
	MINSA	Between 46 and 50	No

*Although they had ESSALUD or MINSA, the interviewees stated that they most frequently use private providers.

** Chronic diseases or long-term treatments.

In the following section, we elaborate on the factors that appear to influence the doctor–patient interaction and how this interaction is perceived by users, including how it affects their perception of the service provided.

### Factors that determine the doctor–patient interaction

From the participants´s perspective, the interaction between patients and doctors is not only influenced by the characteristics or personal attitudes of health providers but also seems to be influenced by contextual elements that affect the way they work and interact with patients. The identified elements have been used to build a conceptual framework to explain this association.

### Insufficiency of human resources (HR)

The interviewees recognized a shortage of HR available for healthcare. From their perspective, this directly affects the services provided: appointments are excessively short and reduced time affects the possibility of addressing all the ailments reported by users. Additionally, participants recognized that this lack of personnel affects not only the time and dedication that can be given to each patient but also how they are treated, considering the fatigue experienced by the providers. Thus, not only is there a shortage of time for care, but also a lack of willingness to address the doubts and inquiries of the users.



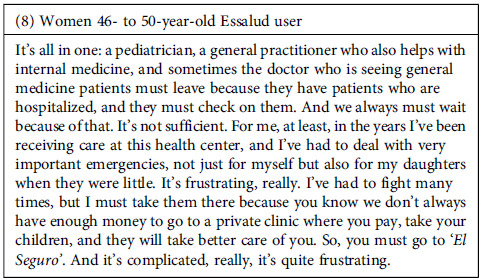



*‘El Seguro’ is a colloquial term frequently used by Peruvians to refer to the healthcare facilities that belong to EsSalud, the employee health insurance system. This will be the meaning going forward whenever the word is used.



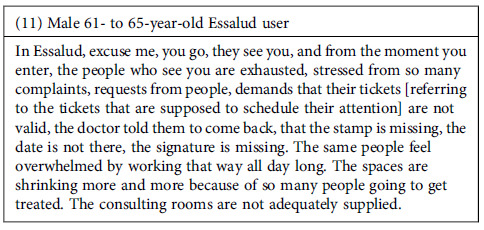



### Contract conditions

Among the interviewees, there was a perception that mistreatment and care failures are, at least partially, a result of bad contract conditions. Some interviewees mentioned being aware of poor working conditions to which healthcare providers are subjected, such as the lack of paid vacations or low salaries. According to them, such elements could explain their lack of enthusiasm for patient care and, in turn, could contribute to the deficiencies in patient care.



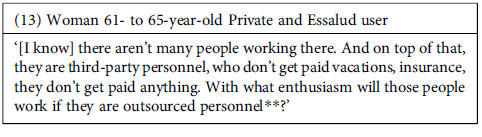



**Outsourced personnel refer to those who do not have a permanent contract but are paid through hourly receipts. This type of personnel is frequently found in all government-funded health services in Peru.

### Infrastructure

Poor infrastructure of healthcare facilities is considered to cause discomfort to the interviewed users and is also an element that affects the healthcare provided. The deficiencies found focus on two aspects: first, the poor maintenance of the infrastructure (walls with cracks or other structural problems), and second, the inadequacy of supplies that are required for health facilities. Regarding the latter, particular mention is made of cleanliness and the availability of basic supplies in restrooms, which are often lacking in toilet paper, soap, and even water. This is not only uncomfortable but could reduce the hygiene of the health centres. Participants also mention inadequate infrastructure, such as small, overcrowded spaces, that can generate a sense of being overwhelmed for both patients and providers.



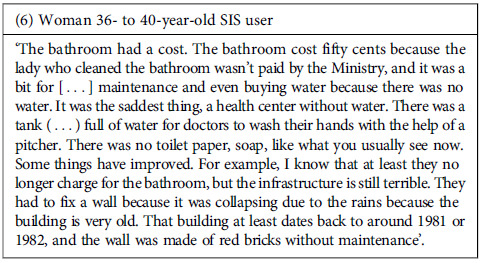





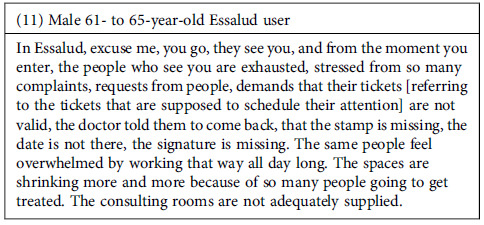



### Patient’s perception of interaction with doctors

The vast majority of participants referred to negative experiences in the health services. In most cases, they perceived a lack of empathy from the health providers, who appeared uninterested in their health problems and needs or seemed to be in a rush during the appointment, trying to finish them quickly.



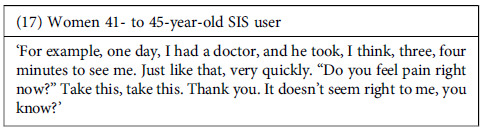





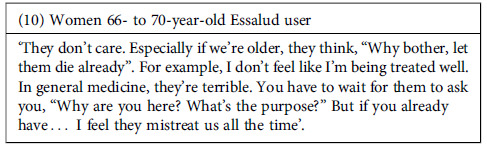



This problem seemed even worse among publicly insured users (public health services provided by MINSA can be covered by SIS insurance or paid for out-of-pocket). According to some testimonies, health providers prioritize the attention of individuals paying out-of-pocket.



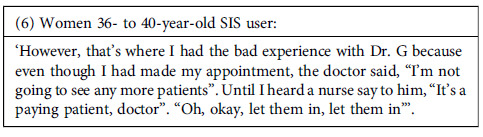



In this scenario, a sense of frustration has grown among some participants. Not only do they feel that they receive poor-quality attention, but they perceive that the system is not designed to address their needs comprehensively, meaning that the care only focuses on addressing a specific ailment without providing further follow-up on their particular case or situation.



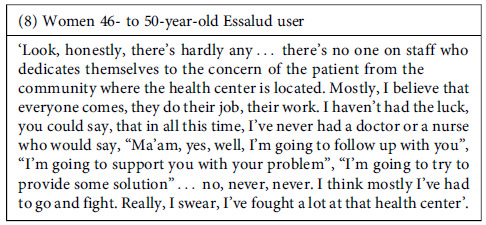



Furthermore, the failures in the system sometimes began with the process that individuals go through to get an appointment. According to the participants, this process was lengthy and cumbersome, must be initiated very early in the morning (with no guarantee of securing an appointment), and was frequently affected by errors in the referral process between services. This problem led to further delays and inconvenience for users. However, despite participants’ frustration with delays in receiving appointments, health providers were intolerant and in the face of any delays that patients might have in attending appointments. Even for reasons beyond the patient’s control, providers were reported to cancel appointments if the patient was not present at the scheduled time.



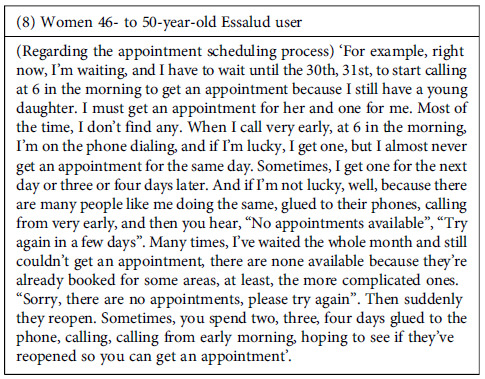


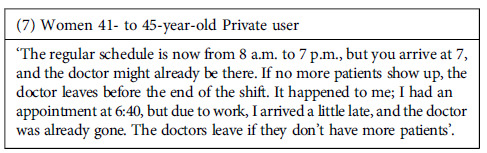



Despite the negative experiences, the perceived effectiveness of the attention might be an element that facilitates patients’ keeping using public healthcare facilities. However, we found that interviewees frequently expressed dissatisfaction with the care they received for their health needs in the facilities they visited. The shortcomings in these experiences revolve around two main aspects: 1. Perception that the care received was inadequate to alleviate their health problems, either because the supplies or medication needed were unavailable or because the providers did not offer them proper treatments. 2. The treatment was poor quality, meaning it was poorly executed or incomplete.



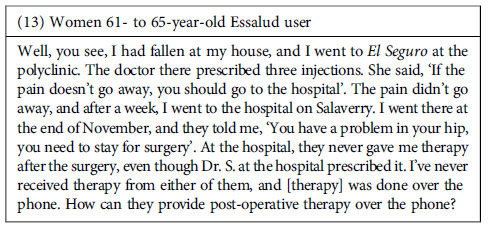





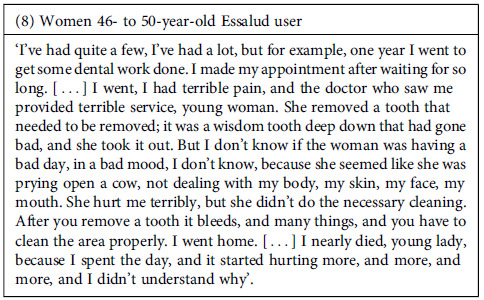



Finally, regarding discrimination based on ethnicity or culture, we did not hear about discriminatory expressions targeting the Afro-Peruvian population. However, discriminatory practices based on ethnic and/or cultural factors were reported, mainly against the Andean population. According to the testimonies, these practices were more frequently associated with non-medical staff than among doctors. These practices included the use of derogatory language against the affected population as well as the low prioritization of their health concerns.



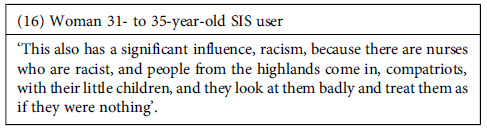



## Discussion

Our study explored the experiences of primary care utilization and the care received in health services among the Afro-Peruvian population in Lima. We conclude that the doctor–patient interaction is not only determined by the personal attitude of health providers but that multiple contextual factors, including human resources, infrastructure, and work conditions all influence this interaction. Additionally, patients’ perception of the interactions is mainly characterized by mistreatment, rushed medical attention, a lack of importance placed on their needs, and a lack of willingness to empathize with and understand those needs. This perception of poor treatment, especially the shortcomings in meeting patients’ needs, is also associated with a perception of low effectiveness of the services.

Our findings align with other studies exploring physician–patient interactions in the primary care system, highlighting deficiencies in care (Smith *et al.*., 2022, Timmins *et al.*., 2022, Little *et al.*., 2015). It is worth noting that these elements are referred to as factors that ultimately affect the treatment provided by healthcare providers, which is the main determinant of satisfaction with the care. This aligns with findings from Switzerland that concluded that treatment and communication, as defining indicators of healthcare quality (Hannawa *et al.*., 2022), often take precedence over structural or outcome-related aspects of care. It also aligns with a Peruvian study that found that the lack of HR results in healthcare providers being overburdened and having little time for each patient (Alarcon-Ruiz, C. *et al.* 2019).

Another factor potentially related to the quality of care and user perception is the infrastructure of healthcare facilities. In Peru, it is estimated that nine out of ten facilities have inadequate infrastructure (Videnza Consultants, 2020). This aligns with our participants’ experiences, who reported that infrastructural deficiencies were primarily evident in the lack of maintenance and basic supplies. It is essential to recognize that, while robust infrastructure is important, it should not be regarded as the sole indicator of quality. In fact, well-equipped facilities may provide substandard care, and the opposite may also occur (Leslie, Sun, & Kruk, 2017). Therefore, negative user perceptions may not solely result from inadequate infrastructure but rather from the interplay of various factors that collectively ensure both quality care and positive patient perceptions.

Additionally, experiences using publicly funded health services are mainly perceived as negative. Patients perceive a lack of interest in their needs, excessive rush in attention, and lack of an integral health approach. Additionally, the attention is perceived as low quality and inefficient in responding to their needs. Moreover, poor disposition from health providers to invest time in attention is even more evident in services not involving direct payments (i.e. those covered by government insurance). This aligns with a past study in Peru (Soto-Becerra, P. *et al.*, 2020), who found that the perception of mistreatment in social security healthcare services (EsSALUD) is associated with belonging to a lower wealth quintile (quintiles 1 or 2, which are indicative of poverty or extreme poverty). It also aligns with a study (Arpey *et al.* 2017), conducted at the University of Iowa Hospital and Clinics in the United States, where individuals believed that their socioeconomic status influenced the quality of healthcare they received, affecting various aspects, including treatment, access to care, and patient–provider interaction. The perception that their care was inferior to that provided to individuals with better socio-economic status frequently eroded trust in the healthcare system.

Our findings on perceived mistreatment within health services among Afro-Peruvians in Lima partially contrast with documented instances of racial discrimination in broader Peruvian society. According to The Group for the Analysis of Development, Afro-Peruvians encounter significant discrimination, particularly in urban areas such as Lima, spanning environments such as public spaces, transportation, workplaces, and educational institutions. These findings underscore the challenges faced by Afro-Peruvians in accessing equitable healthcare (Benavides *et al.*., 2015). However, these estimates contrast with our findings, which revealed no self-perceived explicit instances of ethnic or cultural discrimination directed towards the Afro-Peruvian population within healthcare services. A 2013 report by the Center for Afro-Peruvian Studies and Promotion (LUNDU) (LUNDU, 2013) also did not identify references to ethnic or cultural discrimination targeting the Afro-Peruvian population within healthcare settings. Nevertheless, participants reported an awareness of racial discrimination experienced by their mothers or individuals from earlier generations. It is possible that, over time, healthcare providers have altered such discriminatory behaviours. Nonetheless, it is also plausible that certain discriminatory practices have become normalized in the intervening years.

The main limitation of the research is that the entire studied population is in the province of Lima, experiences in other regions may be distinct. Although Lima was chosen, it is a region with a higher percentage of Afro-Peruvian population. Another limitation is the diversity among sub-health systems, each potentially presenting unique challenges not specifically addressed in our study. Furthermore, the variability in health conditions (acute/chronic) may affect how individuals perceive the healthcare system due to differing impacts of diseases. Despite this, our study is one of the first investigations in Peru that explores the perception of the Afro-Peruvian population regarding physician–patient interaction in first-level public healthcare services. It allows us to identify shortcomings in the healthcare system that need to be overcome and highlights the importance of working on healthcare provider education regarding communication skills for improved patient interaction.

## Conclusions and recommendations

Our findings provide insights for improving healthcare services in terms of quality of care and physician–patient interaction at primary care among the Afro-Peruvian population. This includes empathy, good treatment by healthcare personnel, without discrimination based on socio-economic factors or ethnic group membership, and aspects of healthcare facility infrastructure.

Based on these findings, it is essential to conduct further research within the Peruvian healthcare system to evaluate the best strategies to improve healthcare provider–patient interaction skills. Future studies should explore the aspects of communication that are most important from the patient’s perspective for considering empathetic and compassionate care and what characteristics a curriculum aimed at improving these attributes in healthcare personnel should have.
